# Temperature dependence of emission product distribution from vaping of vitamin E acetate

**DOI:** 10.1371/journal.pone.0265365

**Published:** 2022-03-24

**Authors:** Alexa Canchola, Ruth Meletz, Riste Ara Khandakar, Megan Woods, Ying-Hsuan Lin

**Affiliations:** 1 Environmental Toxicology Graduate Program, University of California, Riverside, CA, United States of America; 2 Department of Environmental Sciences, University of California, Riverside, CA, United States of America; 3 Department of Chemistry, University of California, Riverside, CA, United States of America; University of California, Merced, UNITED STATES

## Abstract

Nearly two years after vitamin E acetate (VEA) was identified as the potential cause of the 2019–2020 outbreak of e-cigarette, or vaping product-associated lung injuries (EVALI), the toxicity mechanisms of VEA vaping are still yet to be fully understood. Studies since the outbreak have found that e-liquids such as VEA undergo thermal degradation during the vaping process to produce various degradation products, which may pose a greater risk of toxicity than exposure to unvaped VEA. Additionally, a wide range of customizable parameters–including the model of e-cigarette used, puffing topography, or the applied power/temperature used to generate aerosols–have been found to influence the physical properties and chemical compositions of vaping emissions. However, the impact of heating coil temperature on the chemical composition of VEA vaping emissions has not been fully assessed. In this study, we investigated the emission product distribution of VEA vaping emissions produced at temperatures ranging from 176 to 356°C, corresponding to a variable voltage vape pen set at 3.3 to 4.8V. VEA degradation was found to be greatly enhanced with increasing temperature, resulting in a shift towards the production of lower molecular weight compounds, such as the redox active duroquinone (DQ) and short-chain alkenes. Low temperature vaping of VEA resulted in the production of long-chain molecules, such as phytol, exposure to which has been suggested to induce lung damage in previous studies. Furthermore, differential product distribution was observed in VEA degradation products generated from vaping and from pyrolysis using a tube furnace in the absence of the heating coil at equivalent temperatures, suggesting the presence of external factors such as metals or oxidation that may enhance VEA degradation during vaping. Overall, our findings indicate that vaping behavior may significantly impact the risk of exposure to toxic vaping products and potential for vaping-related health concerns.

## 1. Introduction

After the 2019–2020 outbreak of e-cigarette or vaping product use-associated lung injury (EVALI) in which the Centers for Disease Control and Prevention (CDC) reported over 2,800 hospitalizations of patients displaying symptoms of acute respiratory distress [1], serious public health concerns have been raised about the safety of e-cigarettes. In the initial investigations, evidence has supported that vaping of vitamin E acetate (VEA), a synthetic form of vitamin E (VE) that was used to “cut” or dilute black market or homemade tetrahydrocannabinol (THC), was a major cause of the onset of EVALI symptoms [1–3]. Several different mechanisms of toxicity have been proposed since the outbreak, yet the exact causative agents and molecular mechanisms through which VEA vaping emissions resulted in lung toxicity are still not well understood.

VE and VEA alone are considered safe for dermatological application in skin-care products [4] and as well as for consumption in foods and dietary supplements [5]. Several studies since the outbreak, however, have found that e-liquids like VEA undergo major thermal decomposition during the vaping process to form products that are often more toxic than the parent oil [6–8]. VEA in particular has been found to decompose into a wide range of emission products including VE, alkenes such as 1-pristene [8, 9], alcohol-containing compounds such as 3,7,11-trimethyl-1-dodecanol, durohydroquinone (DHQ) [6], and durohydroquinone monoacetate (DHQMA) [8, 9], and carbonyl-containing compounds such as ketene [7, 10], 4-acetoxy-2,3,5-trimethyl-6-methylene-2,4-cyclohexadienone (ATMMC) [9], and duroquinone (DQ) [3, 6, 7]. Still, the overall risk of exposure of each identified product to those who vaped VEA is unclear. For example, ketene gas has been hypothesized to form from the cleavage of the acetate group of VEA. However, this reaction has been calculated to only be feasible at temperatures exceeding 500°C–temperatures that are likely to only occur under “dry puff” conditions [10, 11].

The operating temperature of the vape device is one of many parameters–including the model of e-cigarette used, puff duration, interval between puffs, etc.–that a user may alter to customize their vaping experience [12, 13]. A few studies to date have investigated the impact of increased temperature on the size and volume distribution of emitted vaping aerosols, reporting that greater coil temperatures result in larger puff volumes, but decrease the size of emitted particles [14–16]. A recent study in 2021 found that the emission of volatile degradation products, including various carbonyl-containing species, was significantly enhanced when temperature was increased from 170 to 280°C [17]. In addition, increased coil temperature and characteristics of the vape device have also been found to influence other aspects of vaping emissions, such as the release of metals and the level of carbonyl-containing compounds or radical species [16, 18, 19]. E-cigarette atomizers and heating elements are often comprised of various transition metals including nickel, iron, and chromium [20, 21] which not only pose a risk of metal toxicity to vape users [22], but may play a role in the catalysis of thermal degradation of the e-liquid. One study by Saliba et al. (2018) found that e-cigarette filament wires had a significant impact on the production of carbonyl-containing compounds from propylene glycol (PG) vaping, lowering the temperature required to form carbonyl species by nearly 200°C [23]. However, the factors affecting the chemical composition of e-cigarette degradation products have yet to be fully characterized.

The objective of this study was to examine the influence of variable temperature on the product distribution of e-cigarette vaping emissions, using VEA as a model e-liquid. To do so, we performed a non-targeted analysis of the aerosol-phase constituents at relevant, mid-range vaping temperatures using gas chromatography/mass spectrometry (GC/MS). We hypothesized that elevated temperature of the heating coil during vaping could enhance thermal degradation of VEA, causing a shift in emission product distribution and toxicity in vapers. VEA vaping emissions were produced at coil temperatures ranging between 176 to 356°C using a variable voltage vape pen and analyzed using GC/MS with electron ionization (EI) to assess how emission product identity and concentration changes as a function of temperature. In addition, pure pyrolysis of VEA without the influence of the device was also investigated using a tube furnace to investigate potential catalysis by the device itself. The results from this study contribute to our current understanding of the toxicity mechanisms underlying VEA vaping emissions and have significant implications for the potential health risks associated with the use of other e-liquids.

## 2. Materials and methods

### 2.1 Materials

DL-alpha tocopherol acetate (VEA, > 97%), DL-alpha tocopherol (vitamin E, > 97%), tetramethyl-1,4- benzoquinone (DQ, > 98%), durohydroquinone (DHQ, > 95%), 2-methyl-heptene (> 98%), trimethylhydroquninone (> 98%), and phytol (> 95%) were purchased from Tokyo Chemical Industry (TCI America, Inc.). 1, 3, 5-trichlorobenzene (TCB, 98%) was purchased from Alfa Aesar. Acetonitrile (ACN, 99.95%) was purchased from Fisher Chemical.

### 2.2 Temperature measurement

A pen-style e-cigarette battery (Vapros Spinner II, 1650 mAh) was used as a model variable voltage e-cigarette for this study. This vape pen has set nominal voltages of 3.3, 3.8, 4.3, and 4.8 V. These voltages were confirmed using a multimeter to measure the actual voltage of the battery upon activation.

The set-up of the temperature measurements can be seen in S1 Fig in S1 File. The protocol for the thermocouple measurement of the e-cigarette coil and oil temperatures was adapted from Chen et al [11]. To measure the temperature at each voltage setting, the pen was connected to a fresh cartridge (CCell TH2; 0.5 mL, 2.2 Ω) that was filled with VEA standard oil until the oil level sat just above the atomizer base. The oil level in the cartridge was kept consistent between each reading, as the amount of oil in the cartridge has been previously shown to affect the temperatures the coil may reach [11, 18]. Three 1 mm grounded k-type thermocouple wires (MN Measurement Instruments) were connected to a 4-channel data logger (Mo. SDL200; Extech). One thermocouple was kept suspended to measure the temperature of ambient air as a device control. The second thermocouple was inserted into the air flow tube of the cartridge and allowed to rest on the surface of the ceramic coil. This position was chosen to record temperature across all voltage settings as it not only provided the most consistent measurements, but certain positioning of the probe resulted in the battery shutting off, likely to prevent overheating or burning in the event of the air flow tube being blocked during real-use scenarios. The third thermocouple was inserted into the glass casing of the cartridge to submerge the end of the probe in VEA oil in contact with the atomizer. The thermocouples allowed for simultaneous measurement of the coil and the parent oil in the cartridge when the battery was activated. Temperatures were recorded by the data logger every 1 s over a 1 min cycle. The vape pen was activated by holding the power button for 4 s to heat the coil, then allowed to rest for the remainder of the cycle. A total of 13 cycles–including 3 initial preconditioning cycles–were measured.

### 2.3 E-cigarette collection

The procedure for collection of VEA vaping emissions at each temperature setting was adapted from previous studies [6, 7]. Prior to each collection, a fresh cartridge was filled with VEA standard oil, weighed, and preconditioned by taking 3–5 puffs. The vaping emissions were collected using a cold trap apparatus maintained at -40°C (Across International LLC). The particle collection efficiency of the cold trap system at the flow rate used in this study has been reported previously (≥ 99% by volume) [24]. To collect aerosol emissions, one 4 s puff was taken at intervals of 1 min to maintain consistency with the temperature measurement procedure. Puffs were generated at each temperature using a 0.4 L min^-1^ air flow rate, which was controlled by a 0.46 L min^-1^ critical orifice connected a diaphragm pump (Gast Manufacturing Inc.). For each setting, the vape pen was operated until approximately 100 mg of VEA had been consumed; this consumption was typically achieved within 10–20 puffs. In instances where more puffs were required, the vape pen was allowed to rest at 20 puffs for 10–20 minutes to prevent overheating of the battery.

Condensed emission products were dissolved in 1 mL of ACN, with 10 μL of 1, 3, 5-TCB solution (2 μg μL^-1^) added to each sample as an internal standard for chemical analysis. Emissions were analyzed immediately after collection or stored at -80°C to prevent any aging effects.

### 2.4 Tube furnace experiments

To determine the impact of the device on the degradation of e-liquids, pure pyrolysis of VEA oil was simulated using a tube furnace reactor system (OTF-1200X; MTI Corporation). The schematic of the set up for these experiments is shown in S2 Fig in S1 File. An alumina crucible containing 100 mg of VEA standard oil was weighed, and then placed into a high temperature quartz tube furnace capable of reaching temperatures as high as 1200°C. The tube furnace was initially set to 23°C, then ramped to each temperature setting (176, 237, 322, or 356°C; corresponding to the measured coil temperatures described in section 2.3) at a rate of 10°C min^-1^, and then held at the target temperature for 75 minutes to allow for VEA oil to be evenly heated. Inert argon gas was flowed through the system at a rate of 0.18 L min^-1^ (controlled by a critical orifice) to carry the VEA pyrolysis products into cold trap apparatus kept at -40°C. After 75 minutes, the tube furnace was programed to return to room temperature before the alumina crucible was removed and re-weighed to determine the amount of VEA that was consumed. Pyrolysis products condensed in the cold trap were dissolved in 1 mL of ACN and concentrated to 100 μL using a gentle N_2_ gas stream. Then, 10 μL of 1, 3, 5-TCB solution (2 μg μL^-1^) was added to each sample as an internal standard for chemical analysis.

### 2.5 GC/MS analysis of vaping emissions

VEA decomposition products were identified and quantified using GC/MS (Agilent 6890N GC and 5975C inert MSD equipped with an EI ion source) analysis. Large molecular weight and non-polar degradation products were analyzed by directly injecting 2 μL of sample into an Agilent J&W DB-5MS column (30 m × 0.25 mm i.d., 0.25 μm film) for separation. A solvent delay of 6 min was used; the GC was initially set to 60°C for 1 min, then ramped to 150°C at a rate of 3°C min^-1^, held at 150°C for 2 min, ramped to 310°C at a rate of 20°C min^-1^, and then held at 310°C for 5 min. Smaller molecular weight, polar degradation products were analyzed by directly injecting 2 μL of sample into a Rtx-VMS fused silica column (30 m × 0.25 mm i.d., 1.4 μm film). A solvent delay of 6 min was used. The GC was set to 35°C for 1 min, ramped to 240°C at a rate of 10°C min^-1^, and held 4 min. The detailed procedures for the operation of GC/MS can be found in a previous publication [25].

### 2.6 Identification of emission products

Degradation products were identified using the NIST 2008 mass spectral database. Compounds with probability ≥ 50% and match factor scores ≥ 800 were considered as good matches [26, 27]. For compounds that were suspected to be present in our spectra but could not be identified using the NIST library due to lack of available standards, Quantum Chemistry Electron Ionization Mass Spectrometry (QCEIMS) was used to simulate theoretical EI mass spectra of molecules [28]. The detailed procedures for QCEIMS calculations can be found in the supporting information. Peak abundances were normalized to the 1,3,5-TCB internal standard for quantification.

## 3. Results and discussion

### 3.1 Temperature measurement

**Fig 1:**
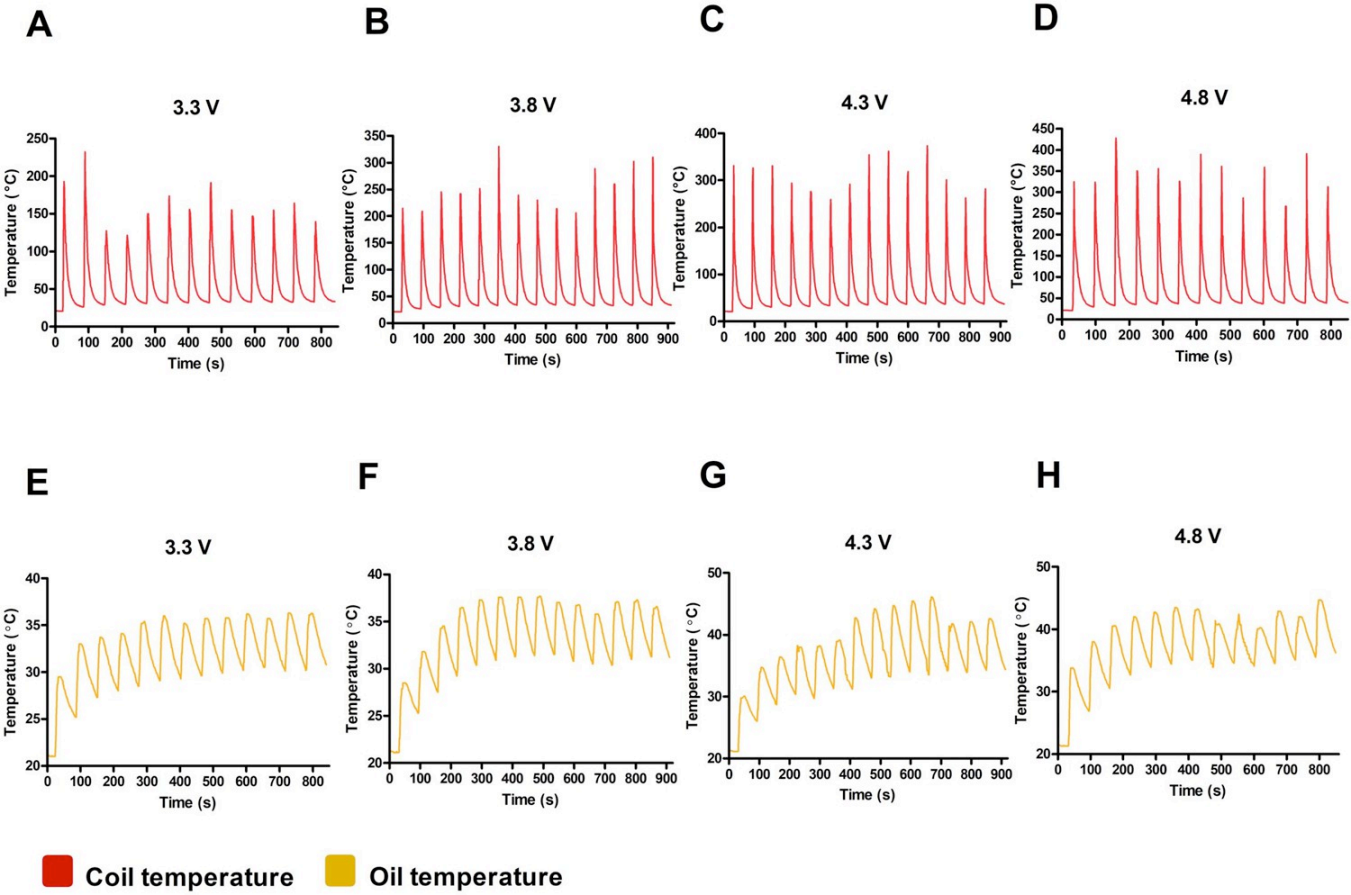
E-cigarette temperature profiles. Temperature profiles of (A–D) e-cigarette coil at 3.3, 3.8, 4.3, and 4.8 V, and (E–H) VEA oil in contact with the atomizer tube at 3.3, 3.8, 4.3 and 4.8 V. Measurements were taken every 1 s over a 1 min cycle; the battery was activated to heat the coil for 4 s, then the pen was allowed to rest for the remaining time.

Fig 1 shows the temperature profiles of the e-cigarette coil (Fig 1A–1D) and VEA oil in the cartridge (Fig 1E–1H) operated at each voltage setting. Peak coil temperature at each voltage setting was fairly consistent between each measurement with no significant increase after consecutive use, which agrees with previous reports [11]. Though the starting temperature after 1 min of rest increased slightly with subsequent measurements, the starting temperature never exceeded 33°C. In contrast, the temperature of the oil in the cartridge increased with each subsequent measurement until seeming to plateau.

**Fig 2:**
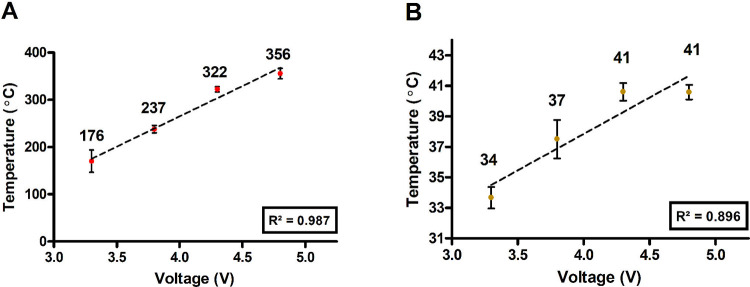
Average peak temperatures vs. voltage. Linear graph of (A) coil and (B) oil average peak temperatures versus each voltage setting of the e-cigarette device.

The peak temperatures of both the coil and the oil were then taken and plotted as a function of voltage, as shown in Fig 2. Coil temperature showed a strong positive linear relationship with applied voltage (Fig 4; R^2^ = 0.987), whereas oil temperature increased linearly with voltage until 41°C (Fig 2B), where the peak temperatures at 4.3 and 4.8 V do not significantly differ. This is likely due to the specific heat capacity of VEA [29]; at higher voltages. Visible discoloration to the oil and wick could be seen during temperature measurements, indicating that the specific heat capacity of the oil in the cartridges may have been exceeded and part of the stored VEA may have been transformed before it is vaped (S3 Fig in S1 File).

### 3.2 Temperature dependence of emission product distribution

**Fig 3:**
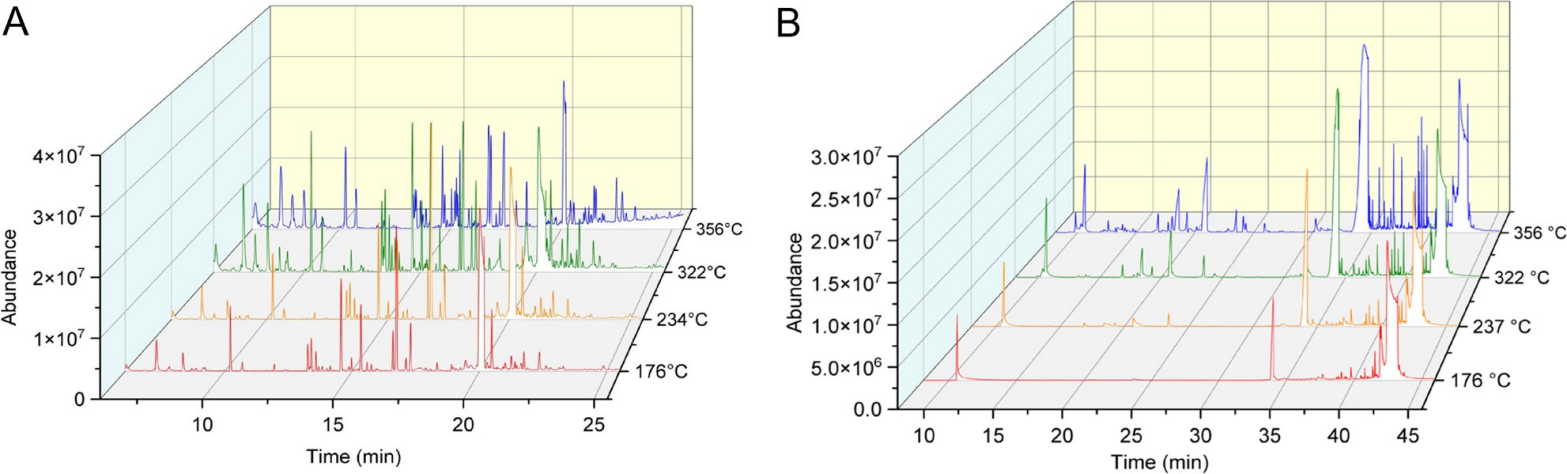
Total ion chromatographs (TIC) of VEA vaping emissions collected at 176, 234, 322, and 356°C. TIC obtained from (A) a polar Rtx-VMS fused silica separation column and (B) a non-polar J&W Scientific DB-5MS separation column.

The total ion chromatographs (TIC) obtained from GC/MS analysis of VEA vaping emissions produced at each temperature setting are shown in Fig 3. Overall, clear temperature dependent degradation of VEA vaping emissions can be seen as the amount and abundance of degradation products substantially increases with increasing coil temperature.

Analysis of the GC/MS results revealed 19 compounds that were able to be tentatively identified based on consistent NIST MS spectral library match scores of 800 or greater. One other compound, 1-pristene, was not found in the NIST library and thus was identified based on comparison with previously reported mass spectra [8] and a mass spectrum generated with the QCEIMS program that found signature fragments of *m/z* 266, 111, and 126, which are consistent with our results (S4 Fig in S1 File). A summary of the identified compounds and chemical information identified from PubChem [30] can be found in the supporting information (S1 Table in S1 File). Many of the products described here, such as phytol, 2,3,5-trimethyl-1,4-benzenediol and 2-hydroxy-4-methoxy-3,6-dimethyl benzaldehyde, have not been previously detected from VEA vaping to our knowledge. An isomer of 2,3,5-trimethyl-1,4-benzenediol has also recently been identified as a substantial VEA degradation product at temperatures ≥ 220°C [17]. Authentic standards were purchased for 2-methyl-1-heptene, phytol, and 2,3,5-trimethyl-1,4-benzenediol to confirm identities of observed products (S5–S7 Figs in S1 File, respectively). Other compounds, such as vitamin E, DQ, DHQ, 1-pristene, and 3,7,11-trimethyl-1-dodecanol, have been consistently identified as VEA decomposition products [3, 6–9]. Several products, such as DHQMA [9] or ketene [7], that have been previously reported in VEA vaping emissions could not be found in our spectra, likely due to the limitations of the emission collection and analysis method described in section 3.4.

**Fig 4:**
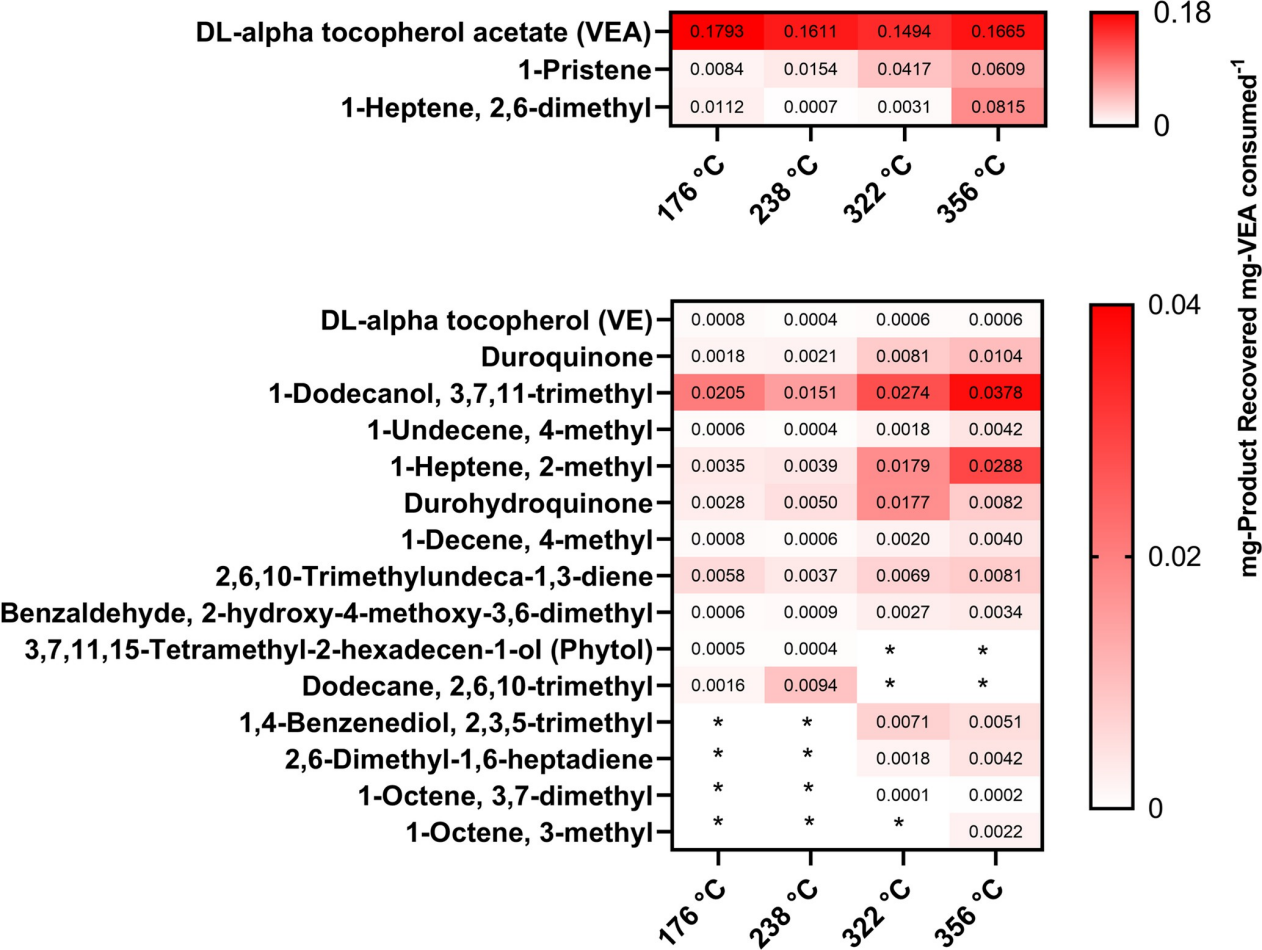
Heatmap of VEA vaping emission product distribution at 176, 237, 322, and 356°C. Compounds were identified via NIST mass spectral library and included based on frequency and consistency of detection throughout all collections. Asterix indicates that the concentration of a product was below the detection limit of the instrument.

A heatmap of the mass fractions of degradation products generated at each temperature is shown in Fig 4. Products that contribute to the majority of the observed VEA degradation (mass fractions ≥ 0.05) were separated from the total heatmap to better visualize the change in each concentration as a function of temperature. VEA, 1-pristene, and 3,7,11-trimethyl-1-dodecanol were found to be the most dominant vaping emission products at all of temperature settings, while other compounds, such as duroquinone, durohydroquinone, and 2-methyl-1-heptene steadily increase in concentration as temperature increases. Furthermore, certain compounds including 2,3,5-trimethyl-1,4-benzenediol, 2,6-dimethyl-1,6-heptadiene, 3,7-dimethyl-1-octene, and 3-methyl-1-octene are not produced in concentrations above the detection limit of our instrument until 322°C, which suggests a potential risk that users who operated vaping devices at lower temperatures would not be exposed to. However, while most identified compounds appear to increase in concentration as temperature increases, phytol and 2,6,10-trimethyl-dodecane are produced at detectable levels at 176 and 237°C but cannot be found at higher temperatures. Another recent study has also detected production of phytol when vitamin E were heated in a microchamber/thermal extractor at 250°C [31]. It is possible that at these compounds are stable at lower temperatures but begin to break down into degradation products themselves as the temperature increases.

Another important pattern to note is the increase in compounds that may pose a risk of oxidative damage to lungs, such as DQ and 2,3,5-trimethyl-1,4-benzenediol, at higher concentrations. While not investigated in this study, prior research has shown that increased temperature may result in the enhanced emission of carbonyl-containing compounds when vaping e-liquids containing propylene glycol and glycerin (PG and VG) [16, 18]. Thus, vaping VEA at greater temperature settings may also carry the risk of exposure to highly electrophilic molecules and subsequent oxidative lung injury.

**Fig 5:**
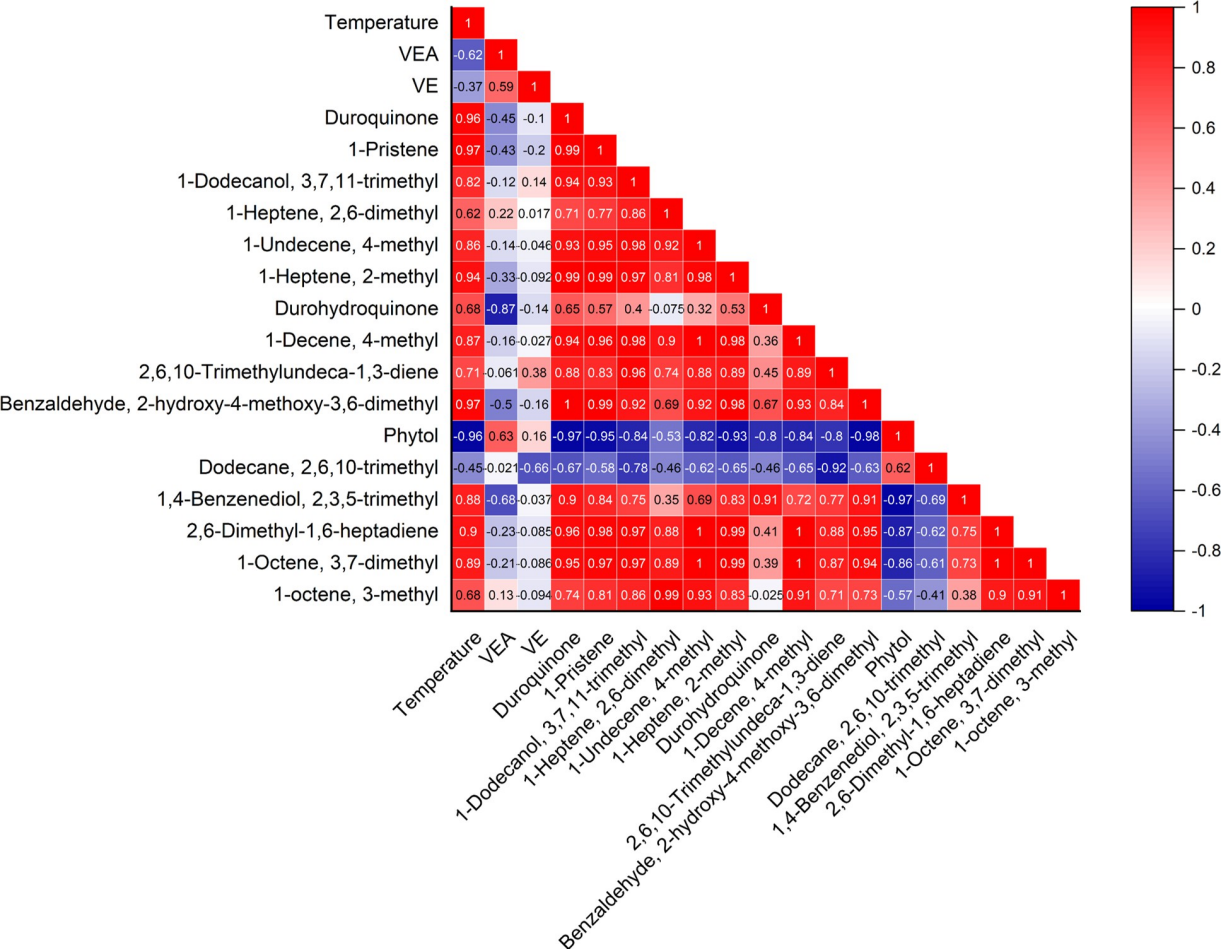
Correlation matrix for production VEA degradation products and temperature. Pearson correlation analysis results depicting interactions between temperature and VEA degradation, and interactions between concentrations of degradation products. Positive correlations (R > 0) are depicted in red, while strong negative correlations (R < 0) are depicted in blue.

In order to better understand the interactions between temperature and the generated emission products, a Pearson correlation analysis was performed (Fig 5). Overall, all but four of the identified compounds were strongly correlated with temperature (R ≥ 0.6). Compounds such as DQ, 1-pristene, 2-methyl-1-heptene, 2-hydroxy-4-methoxy-3,6-dimethyl benzaldehyde, and 2,6-dimethyl-1,6-heptadiene, were very well correlated with temperature (R ≥ 0.9), indicating a strong increase in concentration as temperature increases. VEA and phytol, in contrast, were strongly anti-correlated with temperature (R ≤ -0.6), while VE and 2,6,10-trimethyl-dodecane were moderately anti-correlated with temperature (R ≤ -0.37). In addition, VEA was found to be weakly to strongly anti-correlated with all degradation products excepting phytol and VE, which demonstrate a strong positive correlation (R > 0.5). These results support our analysis of the mass fractions, indicating that as temperature increases, thermal decomposition of VEA is heightened.

Further analysis of the correlations between degradation products shows that phytol is strongly anti-correlated with all VEA degradation products (R < -0.8) with the exception of 2,6,10-trimethyl-dodecane, which was found to have a strong positive correlation with phytol (R = 0.62). Phytol was also found to be strongly correlated with VEA (R = 0.63), likely because as more VEA was evaporated during the vaping process, the greater the chance of degradation into phytol. These relationships further suggest that while some degradation products may be stable at high temperatures, phytol may further decompose into shorter-chain alcohols, alkanes, and alkenes and enhance the production of VEA vaping emission products. Phytol is known both as a precursor for the synthesis of VE and vitamin K12 [32, 33], as well as a byproduct of chlorophyll degradation [33, 34]. Inhalation of aerosolized phytol has previously been shown to induce lung injury in exposed rats [35, 36]. In addition, phytol is a long chain alkyl alcohol compound, meaning that it has the potential to induce damage to the membrane of cells in a biological system [37, 38]. Overall, the toxicity of phytol raises questions about the safety of vaping not only VEA but cannabis-containing vape products that may result in phytol production.

These results clearly indicate that the product distributions of VEA vaping emissions are highly dependent on the operating temperature of the vape pen. As a result, the exposure for vape users operating the same e-cigarette products at different temperatures may differ significantly.

### 3.3 Potential catalysis of VEA vaping pyrolysis

Previous reports of VEA pyrolysis indicate that VEA begins to degrade starting at ~200–240°C [39, 40]. However, our results clearly demonstrate degradation of VEA and formation of products such as DQ at 176°C, indicating that the device itself may play a larger role in the decomposition of VEA than initially anticipated. Previous study in our lab has also found substantial formation of DQ at 218°C–several hundred degrees lower than what has been predicted [24]. To further understand if the device itself may impact the thermal degradation of VEA, pure pyrolysis of VEA oil was carried out using a tube furnace reactor. After 75 minutes, the average mass loss of VEA heated at 176, 237, 322, and 356°C was found to be 0.11 ± 0.091, 0.37 ± 0.11, 3.7 ± 0.072 and 7.1 ± 0.0016 mg of VEA consumed. At 176 and 237°C, VEA was fairly stable; substantial consumption of VEA oil was not observed until the two higher temperatures, despite clear consumption at all temperatures during the vaping collection.

**Fig 6:**
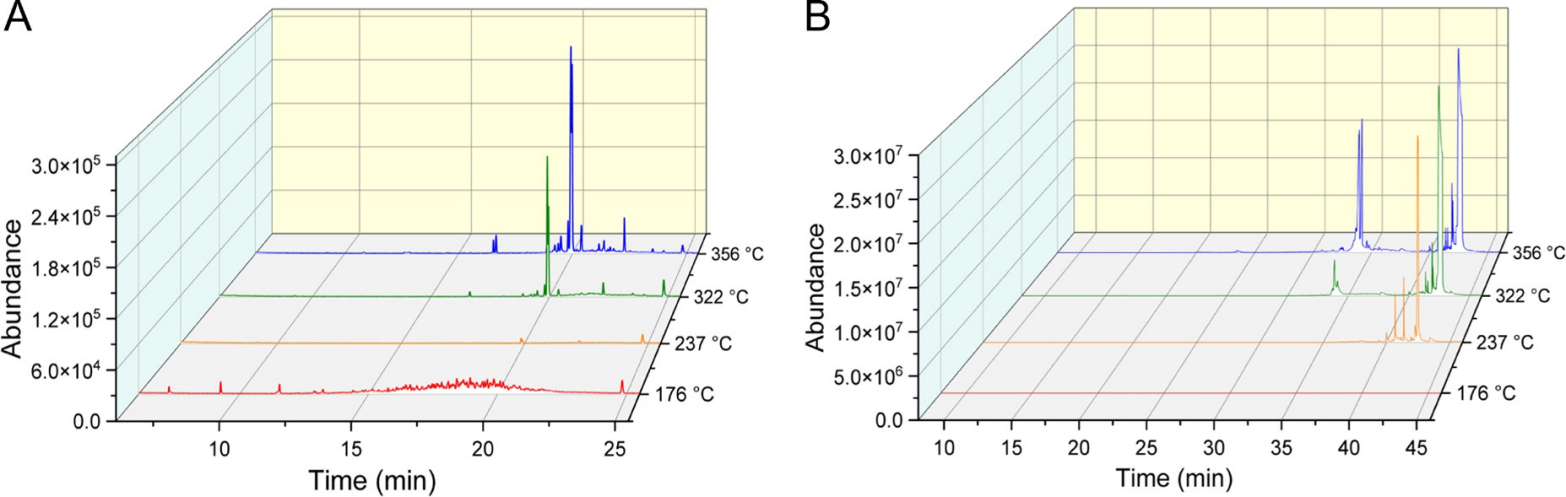
TIC of tube furnace pyrolysis emissions collected at 176, 234, 322, and 356°C. TIC of tube furnace pyrolysis emissions collected at 176, 234, 322, and 356°C obtained from (A) a polar Rtx-VMS fused silica separation column and (B) a non-polar J&W Scientific DB-5MS separation column.

Fig 6 demonstrates the product distribution of VEA degradation products collected and analyzed using GC/MS. Here, we did not observe substantial thermal decomposition of VEA when heated at 176°C for 75 minutes, which greatly contrasts with the degradation of VEA at 176°C for only 4 s during the vaping collection. At 237°C, the parent VEA molecule was the only detectable emission product, indicating that VEA again did not degrade at this lower temperature, though 237°C was enough to evaporate VEA so that it could be collected in the cold trap. Degradation products were only detectable from samples collected at 322 and 356°C, though the number of products and abundance of observed peaks are drastically reduced when compared to the vaping emissions. It should be noted that the tube furnace is capable of heating VEA at more accurate and consistent temperatures than the vape pen itself, which often saw temperature fluctuations that may influence results.

The stark difference in product distribution provides evidence that VEA vaping emissions may not be the result of pure pyrolysis alone. Instead, external factors such as the device elements themselves or environmental interactions may play a role in the catalysis of VEA degradation. The cartridge used in this study is a newer THC cartridge that contains a ceramic heating element, a nichrome filament wire, a fibrous wick/insulation wrap through which oil was delivered to the heating element, and a stainless steel air flow tube and heating element housing that the oil remained in direct contact with [20]. The emission of metals during the vaping process has been documented in several prior studies [21, 41–43], but the interaction between VEA and the metal components of the vape device are still being investigated. Saliba et al. [23] recently found that interaction between a metal heating element and PG greatly decreased the temperature required to observe PG thermal decomposition. Certain metals such as stainless steel, which is present in the cartridge used in this study, resulted in a nearly 300°C reduction in required temperature compared to pure pyrolysis, highlighting a clear interaction between the PG decomposition and the device itself.

Furthermore, a study by Jaegers et al. [44] found that pyrolysis alone in an anaerobic environment was not able to induce thermal degradation of PG and VG at low temperatures (< 200°C), despite previous studies observing degradation at temperatures as low as 149°C during vaping [45]. However, when heated in an aerobic environment, thermal decomposition was observed at 133 and 175°C, both without and with the addition of metal oxides Cr_2_O_3_ and ZrO_2_ [44], suggesting that oxidation is a key process during vaping. In combination with the results shown here, evidence highly suggests that pure pyrolysis alone may not be the only pathway for VEA degradation. During the vaping process, not only may VEA come into direct contact with metals that are present in the filament wire or stainless-steel body, but VEA must also come into contact with molecular oxygen in ambient air. These interactions may promote VEA degradation at temperatures lower than predicted under pure pyrolysis conditions. Ultimately, it is then possible that compounds such as DQ or ketene may be able to form at lower temperatures than what is theoretically calculated if these interactions are considered. However, further study is required to fully understand the effects of the e-cigarette device and vaping environment on the degradation of e-liquids.

### 3.4 Limitations

There are several limitations to the study presented here that should be noted. First, this study presents a range of decomposition products that were identified using a -40°C cold trap and GC/MS analysis. Approximately 40% of the mass of VEA consumed by the vape pen could be attributed to the compounds identified here. However, compounds with high vapor pressure, such as ketene, that have been previously reported from VEA pyrolysis may not have been efficiently captured using the cold trap method described in this study. This method is expected to better traps particle-phase compounds that are able to condense at -40°C and are stable enough to transfer from the cold trap to collection vials at room temperature and is unable to capture highly volatile or reactive VEA vaping emission products. For example, ketene, which is expected to form during VEA pyrolysis, has an estimated boiling point of -56°C [30] and, as a result, was not expected to be observed in our collection. Furthermore, highly volatile and/or reactive compounds such as ketene and various low molecular weight carbonyl-containing species, etc., often require additional derivatization methods that were not used in this study to be observed using GC/MS [7, 45].

This study was also only able to identify compounds with mass spectra that could be found in the NIST mass spectral library. While PubChem currently reports over 111 million unique chemical structures [30], the NIST library used in this study contains MS fragmentation patterns for only 242,466 compounds [27]. As such, a large portion of the TIC for each collection could not be matched to a known compound (match scores < 600). Furthermore, several peaks were observed that were believed to be co-elution of two or more products, which prevented clear analysis of the fragmentation patterns. Several identified products, such as VEA, may also have multiple isomeric forms that have only slight differences in their retention times and mass spectra that the NIST library matching program is unable to account for. In the case of VEA, all peaks were assumed to be and quantified as the same α form, but it is possible for VEA to exist in α, β, γ, or δ forms. This may be true for other structures identified in this study. The use of QCEIMS to identify products that cannot be found in the NIST database, such as 1-pristene, is a potential avenue for further identification of vaping product emissions [46], though its use for non-target analysis is limited if the researcher does not have a proposed structure in mind to simulate fragmentation. While this study was able to account for ~40% of the mass consumed by the pen during the vaping process, the remaining mass is likely attributable to these uncaptured volatile or reactive products, as well as degradation products that were captured, but unable to be identified at this time.

Finally, the vaping topography used in this study was adapted from previous literature on nicotine vaping and optimized for capture of particles in the cold trap system [24]. Real-word nicotine vape users have been reported to inhale between 50–80 mL/puff at greater flow rates than used in this study [47, 48], whereas parameters for THC-vaping have not been well-characterized at this time [49]. The production yields of VEA degradation products reported in this study could consequently differ for those who vaped at higher flow rates. The temperature dependence of product distribution, however, remains true.

## 4. Conclusion

This study assessed the impact of variable temperature and environmental factors on the distribution of particle-phase VEA vaping emission products. Our results support prior research that as the applied temperature of the e-cigarette coil increases, the identities and concentrations of VEA degradation products change considerably. Higher temperatures greatly promote the decomposition of both the parent VEA as well as larger molecular weight degradation products such as VE, phytol, and 2,6,10-trimethyl-dodecane. Moreover, we observed differential product distributions when VEA was vaped versus when VEA was heated in the absence of the device, suggesting that low temperature pyrolysis observed during vaping may require the presence of a catalyst present in the device or surrounding environment. However, further study is required to fully understand this phenomenon and its effect on VEA degradation. Overall, these results provide evidence that temperature and external factors play an important role in VEA decomposition during the vaping process. As each of these compounds may have different chemical properties and toxicity mechanisms, changes in these vaping parameters may impact the exposure to both active and passive vape users who inhale emission products capable of remaining in the air for longer periods of time [50]. In the case of EVALI symptoms, it is possible that there is no one toxicity mechanism through which VEA vaping emission acts; instead, with a wide range of compounds that could form at different temperatures, multiple pathways may interact to cause damage.

While temperature and the vaping device clearly impact the degradation of the e-liquid used, it is important to note the wide range of customizable vaping parameters including the e-liquids used, the flow rate applied, the puff duration and interval between puffs, and more. Many of these parameters have already been found to significantly impact the degradation of e-liquids used and thus the health risks to vape users [13, 15, 41, 51]. It is very likely that the severity of vaping related lung injuries is dependent on the interactive effects of these customizations; two parameters may synergistically promote degradation of an e-liquid, while others may interact antagonistically to suppress degradation. Further studies are required to fully understand how these varying parameters and external factors may together impact exposure.

## Supporting information

S1 Dataset (ZIP)

S1 File (PDF)

## Abbreviations

EVALI: e-cigarette or vaping associated lung injury
VEA: vitamin E acetate
DQ: duroquinone
DHQ: durohydroquinone
QCEIMS: Quantum Chemistry Electron Ionization Mass Spectrometry
