# Supplementary material for: Temperature dependence of emission product distribution from vaping of vitamin E acetate
Source: PLoS One. 2022 Mar 24;17(3):e0265365. doi: 10.1371/journal.pone.0265365 (PMC8947410; doi:10.1371/journal.pone.0265365)
Supplement: S1 File — (PDF) [file pone.0265365.s002.pdf]

Supporting Information

Temperature Dependence of Emission Product Distribution from Vaping of Vitamin E Acetate

Alexa Canchola[1¶],Ruth Meletz[2¶], Riste Ara Khandakar[2&], Megan Woods[3&], Ying-Hsuan Lin[1,2*]

[1]Environmental Toxicology Graduate Program, University of California, Riverside, CA, USA

[2]Department of Environmental Sciences, University of California, Riverside, CA, USA

[3]Department of Chemistry, University of California, Riverside, CA, USA

[*]Corresponding Author

Email: ying-hsuan.lin@ur.edu

[¶]These authors contributed equally to this work.

[&]These authors also contributed equally to this work

Number of Pages: 11

Number of Tables: 2

Number of Figures: 7

S1. Materials and Methods

S1.1 QCEIMS

The simulated electron impact (EI) mass spectrum for 1-pristene was generated using the Quantum Chemical Electron Ionization Mass Spectra (QCEIMS) program [1, 2]. The molecule of interest was visualized using Gaussview 6. The geometry of the investigated systems was optimized using the DFT/ 6-31G(d) level of theory with a hybrid functional B3LYP. To make the constructed systems interests compatible with QCEIMS, 3-D coordinates were extracted from the optimized (*.log) output files and converted to Turbmole format (*.tmol) format using Openbabel. The cartesian coordinates of the optimized 1-pristene structure can be found in Table S2.

Within QCEIMs, the standalone method GFN-xTB2 method with D4/SV(P) basis set was used for the molecular dynamic calculations. Each system was run with the following parameters: 70 eV ionization energy, 500 K initial temperature, 0.25 fermotsecond time steps with 1425 parallel cluster runs and an impact excess energy (IEE)/atom of 0.6eV. The theoretical spectra were exported using the QCEIMS plotms program and visualized with a python script.

Table S1. Summary of VEA vaping emission products.

Name	Formula	M.W.[a]	CAS #	Structure	EIC	Average NIST Match Score[c]
**DL-alpha tocopherol acetate (VEA)**	$C_{31}H_{52}O_3$	472.7	58-95-7	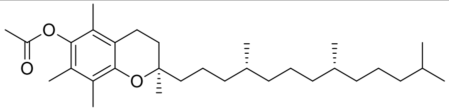	472, 430	902
**DL-alpha tocopherol (VE)**	$C_{29}H_{50}O_2$	430.7	10191-41-0	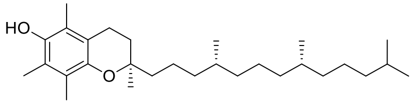	205	895
**Duroquinone**	$C_{10}H_{12}O_2$	164.2	527-17-3	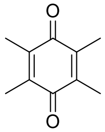	121	892
**1-Pristene[d]**	$C_{19}H_{38}$	266.5	2140-82-1	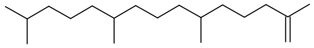	111, 266	N/A
**1-Dodecanol, 3,7,11-trimethyl**	$C_{15}H_{32}O$	228.41	6750-34-1	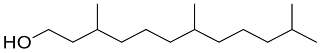	111	856
**1-Heptene, 2,6-dimethyl**	C_9H_{18}	126.24	3074-78-0	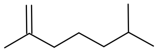	69, 126	848
**1-Undecene, 4-methyl**	$C_{12}H_{24}$	168.32	74630-39-0	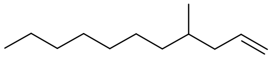	57, 126	800
**1-Heptene, 2-methyl**	C_8H_{16}	112.21	15870-10-7	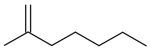	56, 112	801
**Durohydroquinone**	$C_{10}H_{14}O_2$	166.22	527-18-4	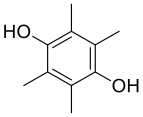	164	890
**1-Decene, 4-methyl**	$C_{11}H_{22}$	154.29	13151-29-6	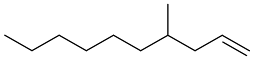	71, 112	839
**2,6,10-trimethylundeca-1,3-diene**	$C_{14}H_{26}$	194.36	20056-22-8	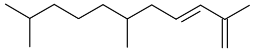	109	817

**Benzaldehyde, 2-hydroxy-4-methoxy-3,6-dimethyl**	$C_{10}H_{12}O_3$	180.2	34883-15-3	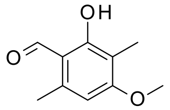	180	823
**3,7,11,15-Tetramethyl-2-hexadecen-1-ol (Phytol)**	$C_{20}H_{40}O$	296.5	150-86-7	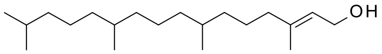	123	804
**Dodecane, 2,6,10-trimethyl**	$C_{15}H_{32}$	212.41	3891-98-3	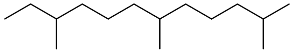	71, 85	832
**1,4-Benzenediol, 2,3,5-trimethyl**	$C_9H_{12}O_2$	152.19	700-13-0	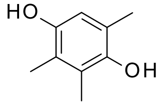	152	836
**2,6-Dimethyl-1,6-heptadiene**	C_9H_{16}	124.22	51708-83-9	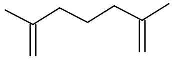	109	876
**1-Octene, 3,7-dimethyl**	$C_{10}H_{20}$	140.27	4984-01-04	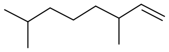	55, 140	853
**1-Octene, 3-methyl**	C_9H_{18}	126.24	13151-08-01	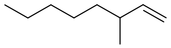	55, 70	813

Summary of compounds identified from VEA vaping emission at each temperature. Information for each compound was obtained from PubChem [3].

[a] M.W.: Molecular Weight (g mol^{-1})
[b] EIC: extracted ion chromatograph; ion selected for quantification
[c] Average match score over all collections
[d] tentative identification based on QCEIMS

Table S2. Cartesian Coordinates for optimized 1-pristene structure calculated by DFT/B3LYP/6-31G(d) level of theory using Gaussian 16W.

C	1.4556	2.5588	-0.4419
C	0.1548	2.5618	0.3875
C	-1.0279	1.8209	-0.2468
C	-3.5385	1.3131	-0.0081
C	-2.2859	1.9557	0.6197
C	2.1424	1.1834	-0.5674
C	2.3946	0.5004	0.7801
C	-3.3939	-0.1882	-0.3362
C	3.0745	-0.869	0.6531
C	1.2248	3.1483	-1.8381
C	-3.1609	-1.0999	0.8754
C	-4.7563	1.5737	0.8849
C	4.4851	-0.8724	0.0339
C	-2.9747	-2.5739	0.4878
C	5.0206	-2.3064	-0.0179
C	5.4639	0.0175	0.8
C	-1.8207	-2.8385	-0.4504
C	-0.443	-2.5224	0.0546
C	-2.0145	-3.3627	-1.6705
H	2.158	3.2241	0.0791
H	0.3402	2.1775	1.3962
H	-0.1409	3.6105	0.5312
H	-0.7624	0.7681	-0.3705
H	-1.2459	2.2263	-1.2402
H	-3.727	1.8254	-0.9616
H	-2.0951	1.5249	1.6099
H	-2.4872	3.0225	0.7836
H	1.5337	0.5206	-1.1952
H	3.0942	1.3243	-1.0927

H	1.4397	0.3266	1.2883
H	2.9791	1.1582	1.4324
H	-4.3073	-0.5205	-0.8474
H	-2.5885	-0.3114	-1.0677
H	3.1233	-1.3144	1.6556
H	2.4275	-1.5257	0.0587
H	0.7095	4.1125	-1.7772
H	2.1829	3.3172	-2.342
H	0.6359	2.4797	-2.4738
H	-4.0164	-1.0369	1.5575
H	-2.2852	-0.7625	1.4402
H	-4.943	2.6496	0.9733
H	-4.6115	1.1804	1.8961
H	-5.6566	1.1152	0.4628
H	4.4258	-0.5093	-0.9988
H	-3.9131	-2.9391	0.0505
H	-2.8218	-3.162	1.4017
H	5.1201	-2.7309	0.987
H	6.0048	-2.3371	-0.4974
H	4.3494	-2.9518	-0.5944
H	5.5127	-0.2643	1.8573
H	5.1748	1.0711	0.7395
H	6.473	-0.0651	0.3818
H	0.3325	-2.8176	-0.6603
H	-0.3302	-1.4514	0.2388
H	-0.2497	-3.0576	0.9899
H	-3.0093	-3.6015	-2.0314
H	-1.1832	-3.5626	-2.3382

11

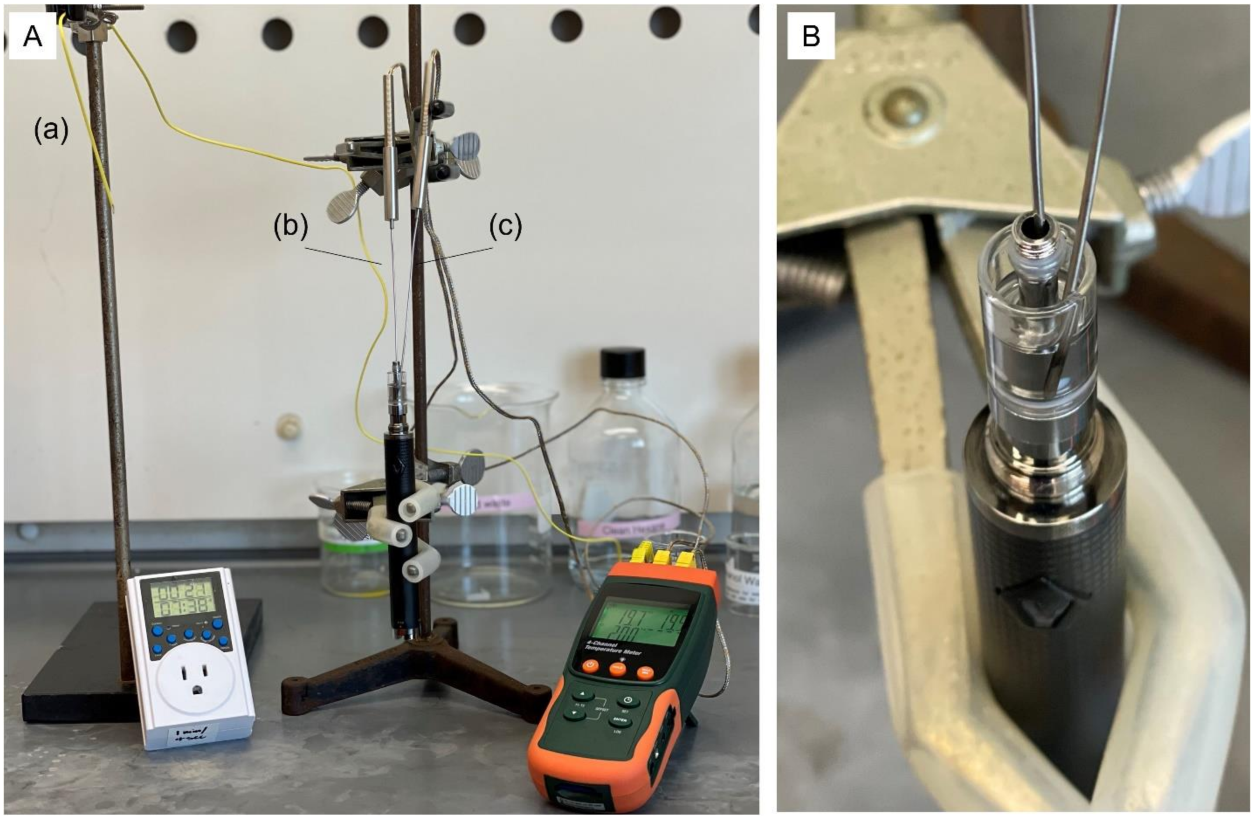

Fig S1. **Set up of e-cigarette temperature measurements.** (A) Set-up of temperature measurements. Set up of e-cigarette temperature measurements. Three k-type thermocouple wires were connected to a data logger, which recorded the temperature of (a) ambient air, (b) the ceramic coil of the e-cigarette cartridge, and (c) VEA oil in contact with the atomizer tube every 1s. (B) Close up of thermocouples inserted into the cartridge.

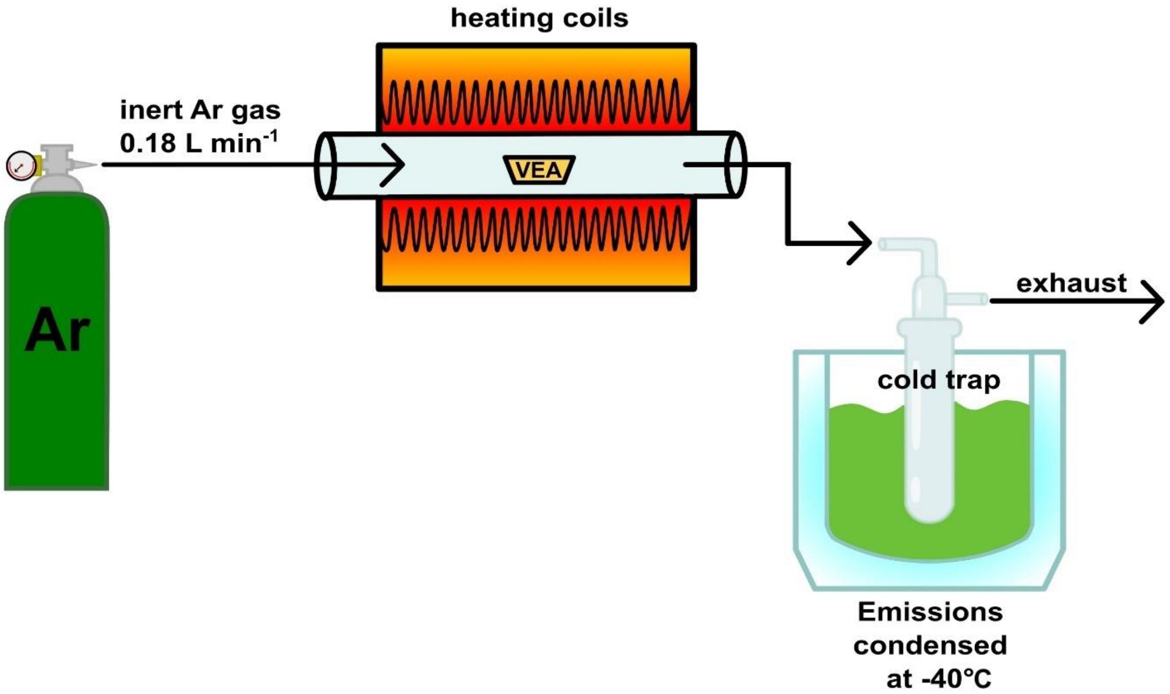

Fig S2. **Schematic diagram of a high temperature quartz tube-furnace system.** Gas flow is regulated by a 0.18 L min^{-1} critical orifice and argon gas is delivered into the quartz tube by a gas tank. Pyrolysis of VEA occurs as the furnace is heated by heating coils and generated aerosol is carried into a cold trap. The exhaust is removed via fume extractor.

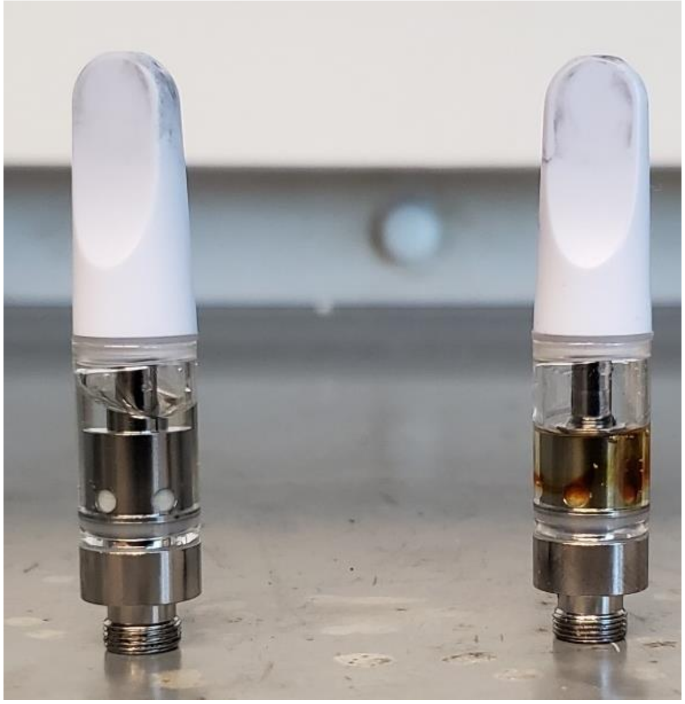

Fig S3. Cartridges heated at 176 and 356 °C. Visible degradation and discoloration could be seen in the cartridge heated at 356 °C (right) versus the cartridge heated at 176 °C (left) after 13 cycles of 4s battery activation during temperature measurements.

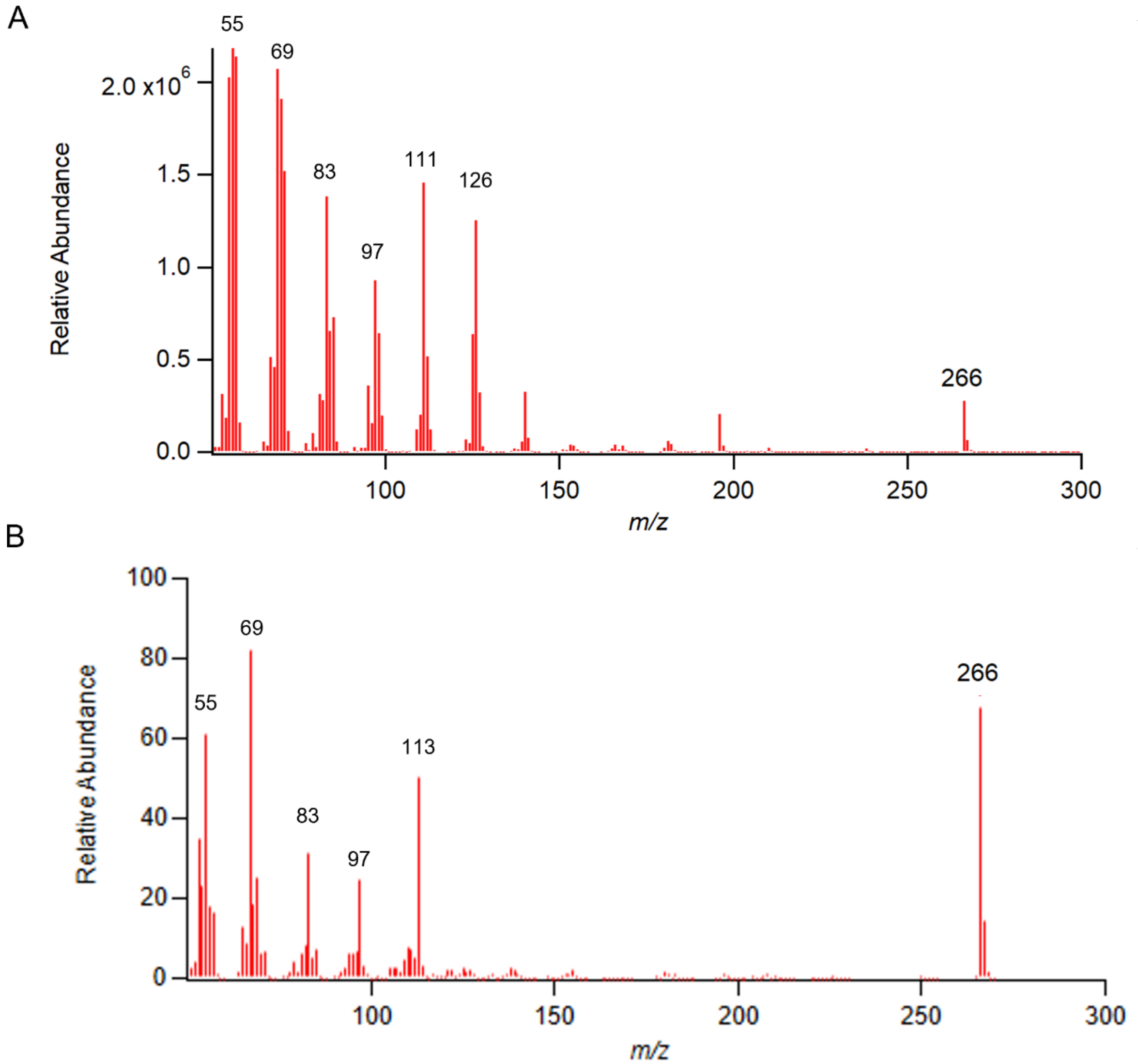

Fig S4. **Comparison of mass spectra for 1-pristene identification**. (A) Experimental mass spectrum obtained from vaping of VEA containing signature fragments with *m/z:* 266 (consistent with the molecular ion of 1-pristene), 111, 126, 97, 83, 69, 55. These identified fragments are consistent with the experimental mass spectrum identified as 1-pristene by Mikheev et al (4). (B) Simulated mass spectrum of 1-pristene obtained using QCEIMS containing signature fragments of *m/z:* 266, 111, 97, 83, 69, and 55.

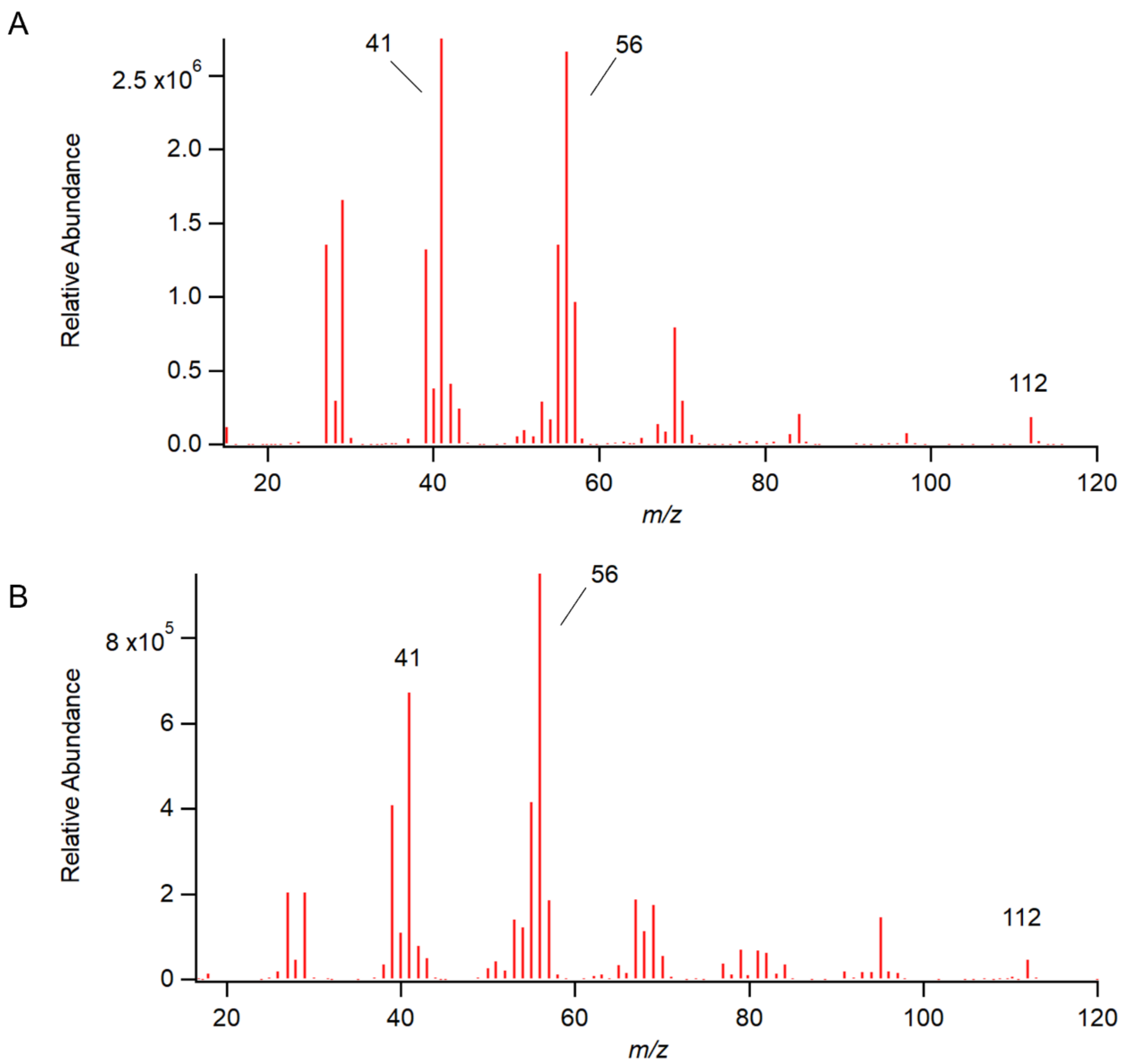

Fig S5. Comparison of mass spectra for 2-methyl-1-heptene identification (A) Experimental mass spectrum obtained from vaping of VEA containing signature fragments with *m/z:* 41, 56, and 112, consistent with 2-methyl-1-heptene. (B) Mass spectrum of authentic 2-methyl-1-heptene standard containing *m/z*: 41, 56, 112.

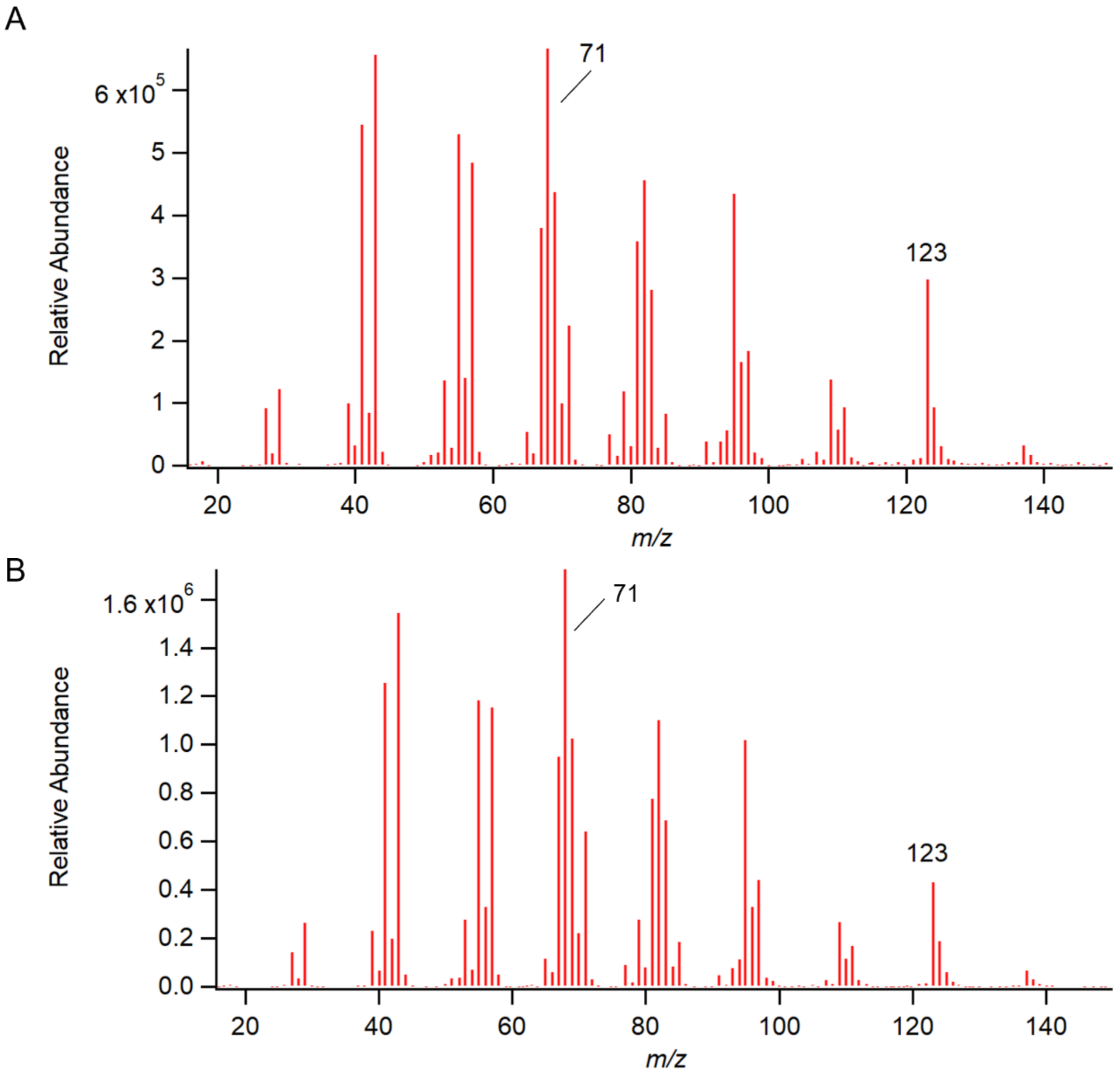

Fig S6. Comparison of mass spectra for phytol identification. (A) Experimental mass spectrum obtained from vaping of VEA containing signature fragments with *m/z:* 123 and 71, consistent with the natural isomer of phytol. (B) Mass spectrum of authentic phytol standard (natural isomer) containing *m/z*: 123 and 71.

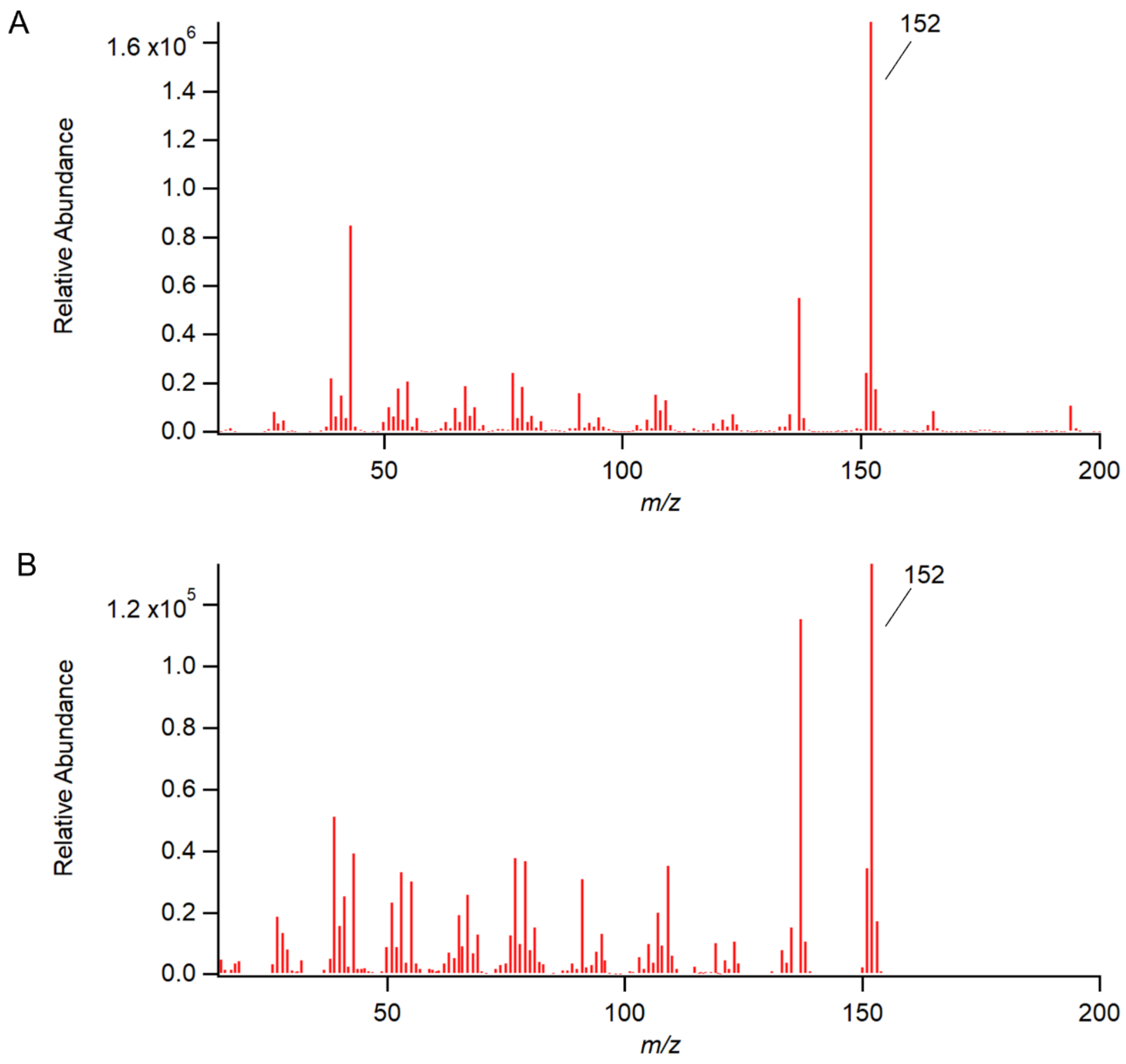

Fig S7. Comparison of mass spectra for 2,3,5-trimethyl-1,4-benzenediol identification. (A) Experimental mass spectrum obtained from vaping of VEA containing signature fragments with *m/z:* 152, consistent with 2,3,5-trimethyl-1,4-benzenediol. (B) Mass spectrum of authentic 2,3,5-trimethyl-1,4-benzenediol standard containing *m/z*: 152.

References

1. Grimme S. Towards First Principles Calculation of Electron Impact Mass Spectra of Molecules. Angewandte Chemie International Edition. 2013;52(24):6306-12.
2. Koopman J, Grimme S. Calculation of Electron Ionization Mass Spectra with Semiempirical GFNn-xTB Methods. ACS Omega. 2019;4(12):15120-33.
3. Kim S, Thiessen PA, Bolton EE, Chen J, Fu G, Gindulyte A, et al. PubChem Substance and Compound databases. Nucleic Acids Res. 2016;44(D1):D1202-D13.
